# Context-explorer: Analysis of spatially organized protein expression in high-throughput screens

**DOI:** 10.1371/journal.pcbi.1006384

**Published:** 2019-01-02

**Authors:** Joel Ostblom, Emanuel J. P. Nazareth, Mukul Tewary, Peter W. Zandstra

**Affiliations:** 1 Institute of Biomaterials and Biomedical Engineering, University of Toronto, Toronto, ON, Canada; 2 The Donnelly Centre, University of Toronto, Toronto, ON, Canada; 3 Medicine by Design, A Canada First Research Excellence Program at the University of Toronto, Toronto, ON, Canada; 4 Michael Smith Laboratories, University of British Columbia, Vancouver, British Columbia, Canada; 5 School of Biomedical Engineering, University of British Columbia, Vancouver, British Columbia, Canada; Hebrew University of Jerusalem, ISRAEL

## Abstract

A growing body of evidence highlights the importance of the cellular microenvironment as a regulator of phenotypic and functional cellular responses to perturbations. We have previously developed cell patterning techniques to control population context parameters, and here we demonstrate context-explorer (CE), a software tool to improve investigation cell fate acquisitions through community level analyses. We demonstrate the capabilities of CE in the analysis of human and mouse pluripotent stem cells (hPSCs, mPSCs) patterned in colonies of defined geometries in multi-well plates. CE employs a density-based clustering algorithm to identify cell colonies. Using this automatic colony classification methodology, we reach accuracies comparable to manual colony counts in a fraction of the time, both in micropatterned and unpatterned wells. Classifying cells according to their relative position within a colony enables statistical analysis of spatial organization in protein expression within colonies. When applied to colonies of hPSCs, our analysis reveals a radial gradient in the expression of the transcription factors SOX2 and OCT4. We extend these analyses to colonies of different sizes and shapes and demonstrate how the metrics derived by CE can be used to asses the patterning fidelity of micropatterned plates. We have incorporated a number of features to enhance the usability and utility of CE. To appeal to a broad scientific community, all of the software’s functionality is accessible from a graphical user interface, and convenience functions for several common data operations are included. CE is compatible with existing image analysis programs such as CellProfiler and extends the analytical capabilities already provided by these tools. Taken together, CE facilitates investigation of spatially heterogeneous cell populations for fundamental research and drug development validation programs.

## Author summary

Cell behavior is influenced by cues that cells receive from their surrounding environment such as signals secreted from other cells and cell-to-cell contact. These factors are spatially heterogeneous and cells at different positions within a colony will experience varying degrees of influence from such environmental cues. In vitro assays often do not allow control over environmental variables and there is a lack of easy to use software to investigate the effect of spatial variation in these factors. We have developed a software package to address this gap and facilitate the quantification of spatially heterogeneous cell responses. Our software accurately identifies colonies of cells within a well and individual cells can be grouped according to their position within these colonies, which enables quantification of cell response as a function of cellular location. To support broad scientific accessibility, the full functionality of the software is available through a graphical user interface. Using this software to analyze data from a screening-optimized micropatterning platform, we show that human pluripotent stem cell-derived colonies grown either under pluripotency maintenance or differentiation-inducing conditions exhibit cell responses that are dependent on spatial organization. This technology should enable more accurate and predictive context-dependent drug screening and cell-fate investigation.

This is a *PLOS Computational Biology* Software paper.

## Introduction

Emerging pieces of evidence stress the importance of a cell’s local microenvironment as a regulator of cellular phenotype and gene expression heterogeneity within cell populations. Microenvironmental parameters such as mechanical forces, cell to cell contact and endogenous signaling, all vary between cells at different positions in a well [1,2]. The spatial heterogeneity of these factors leads to variability in efficiency of endocytosis and the vulnerability to viral infection [3], influences epithelial tissue growth [4], impacts the expression of angiogenic factors in tumor cells [5] and influences the differentiation potential of mouse and human pluripotent stem cells (mPSCs, hPSCs) [2,6]. Microenvironmental heterogeneity is also a potential confounding factor behind contradictory findings in the response of different cell types to key signaling pathway activity [7–9] and could limit the interpretation and reproducibility of experiments.

A comparative analysis [10] of two large scale pharmacogenomic studies, the Cancer Genome Project [11] and Cancer Cell line Encyclopedia [12], revealed a surprisingly poor correlation between cell line drug response phenotypes between laboratories, which prevented meaningful extraction of drug-gene relationships. Correlation remained low even when using matched protocols and cell lines with highly correlated gene expression profiles. Although the exact source of variation in this study is unknown, a separate analysis of single cell data from 45 high-throughput (HTP) screens revealed that population context is indeed a ubiquitous source of variation between screens, and accounting for population context can improve experimental reproducibility between cell lines and laboratories [9]. Although it is acknowledged that understanding population heterogeneity is critical in biomedical research [13,14], the scientific community has been slow to adopt approaches to reduce heterogeneity, such as controlling microenvironmental variables.

The increasing affordability of high content screening instruments, emergence of core screening facilities and technological advancements such as micropatterning in multi-well plates [15], enable investigation of population context dependent variables with unprecedented throughput and veracity [16]. By patterning extracellular matrix (ECM) proteins on a tissue culture surface, cells can be restricted to adhere to an array of spots of predefined shapes and sizes [17,18]. An advantage of such patterning is enhanced control over microenvironmental variation within each well and improved assay robustness [19,20]. Growing cells in colonies of defined size and shape facilitates analyses of inter- and intra-colony variation in protein expression. For example, we observe that hPSCs growing in such patterned colonies express varying levels of pluripotency markers, including SOX2 and OCT4, depending on colony size [2,19]. Elucidating the impact of the population context dependent variables on cellular phenotype will not only add to our understanding of fundamental cell biology, but will also allow us to optimize culture conditions and cell assays, provide possible explanations for current seemingly conflicting research and inform in silico models. These aspects are critical to next-generation drug development strategies and systems biology approaches.

**Fig 1:**
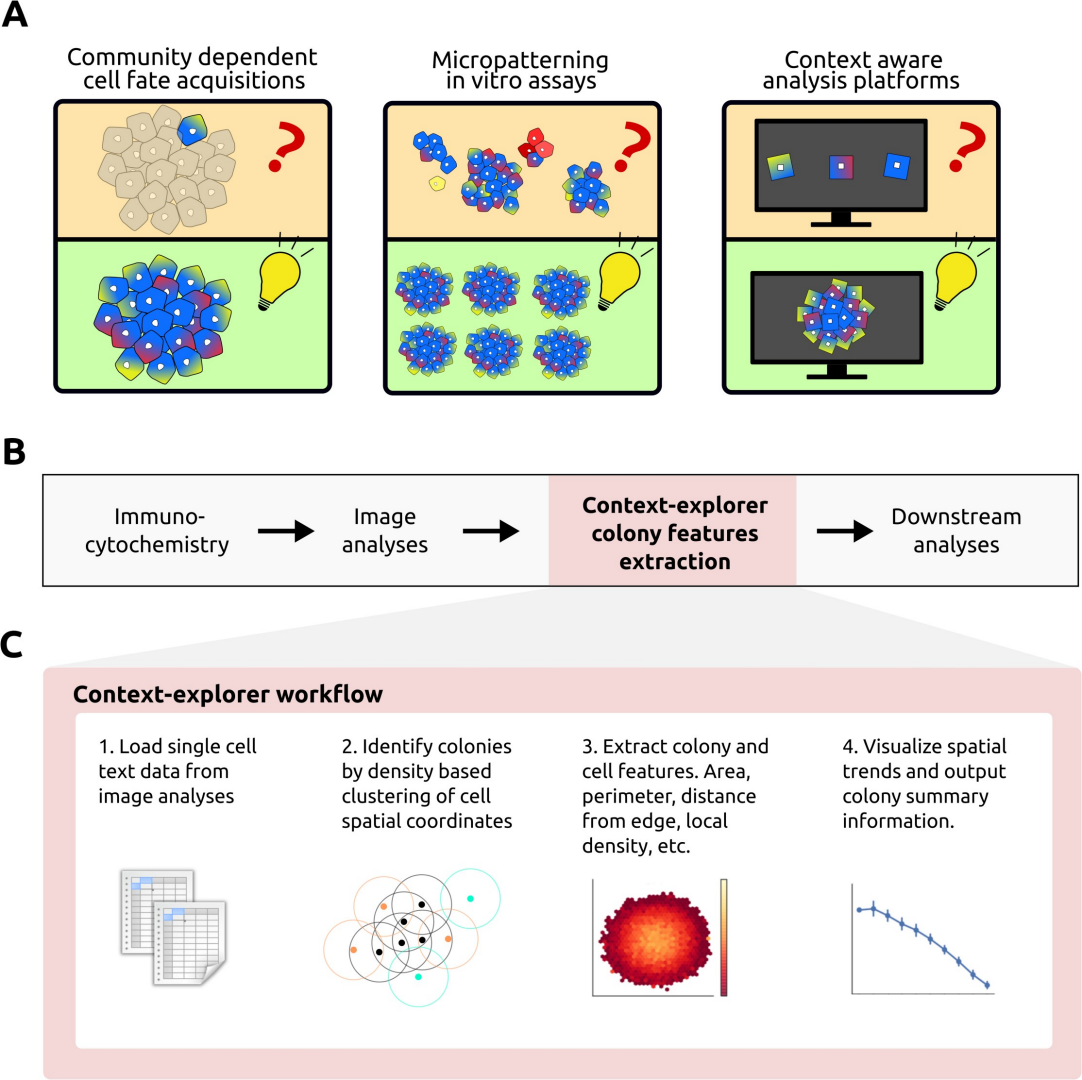
Background schematic and CE workflow overview. **A)** Studying cells in isolation disregards the effects of community interactions, which are known to direct cell fate decisions (*left*). Powerful micropatterning *in vitro* assays increase control over the cellular microenvironment and facilitate the study of context dependent cell fate acquisitions (*middle*). Our analysis software enhances these assays by allowing researchers to analyze cell behavior within its population context instead of as independent isolated events (*right*). **B)** CE fits into existing image analysis pipelines after initial measurements have been extracted from the images. **C)** Overview of the CE workflow, each step is described in detail in the methods section.

We have previously developed an HTP platform for micropatterning of cells on ECM spots of defined shapes in multi-well plates [19,21]. To augment this platform, we here present a computational tool, context-explorer (CE), which facilitates colony level analyses and cell patterning quality control. The CE software is meant to extend the functionality of currently available software solutions, both open source and commercial, for analyzing features of imaged cells [22–24]. While some implementations already exist that can be used to identify arrays of cells on glass slides [25] or to study differential gene expression of cells in different spatial locations within the same colony [26], our software aims to improve the HTP workflow for analysing cells in micropatterned multi-well plates by facilitating evaluation of patterning fidelity, enabling identification of colonies within a well, and improving spatial analyses of heterogeneous protein expression within colonies. As HTP technologies become more widespread, it is increasingly important to provide user friendly data analysis software targeted towards these platforms (**Fig 1A**). Here, we demonstrate the utility of CE by investigating the impact of intra-colony location on hPSC pluripotency marker expression.

## Design and implementation

Designed to complement existing imaging software, CE fits into the analysis pipeline following the extraction of cellular features from microscope images (**Fig 1B**). The input to CE is a CSV-file, which contains single cell xy-coordinates, well label, and at least one other measurement of interest, such as protein fluorescent intensity values. These single cell coordinates can be clustered into colonies within which spatial trends for the measurements of interest can be visualized (**Fig 1C**). By leveraging existing image extraction software and processing the resulting text files, CE has low system requirements and runs smoothly on modern laptop computers. CE is implemented in Python, and utilizes the scientific open source ecosystem SciPy [27]. Specifically, NumPy [28] and Pandas [29] are used for array manipulations, while Matplotlib [30] and Seaborn [31] generate the graphical visualizations. To make CE easily accessible to a broad scientific community of various technical backgrounds, all functionality is available via a graphical user interface designed in Qt.

To interrogate organized cell behavior within colonies, the concept of cellular colonies must first be introduced by classifying closely positioned cells as belonging to the same colony. Manually labeling individual cells is infeasible in HTP assays that often include millions of cells. There are many existing algorithms for automatically identifying dense clusters of data points [32] and CE employs the Density-Based Spatial Clustering of Applications with Noise (DBSCAN) algorithm [33], as implemented in the scikit-learn Python package [34], to identify sets of points at high two dimensional density. Clustering cells into colonies based on local cell density is similar to how these communities are defined biologically since cellular communication is restricted by the distance between cells. The DBSCAN algorithm is capable of identifying colonies of any geometrical shape and performs well on any cell constellation where the distance between neighboring cells within a colony is shorter than the distance between neighboring colonies. DBSCAN scan also has the advantage that it has a notion of outliers, cells far away from any colony, and can classify such cells as noise rather than trying to force all cells to belong to a colony as many other cluster algorithms would do. DBSCAN performs unsupervised clustering and does not require prior knowledge of the number of colonies within each well, only specification of the neighborhood search radius (Eps) and the minimum number of points (MinPts) within the neighborhood to start propagating a cluster. For each point found within the Eps neighbourhood of the starting point, a search for additional points will be performed. If the number of points found within a point’s Eps neighborhood is greater or equal to MinPts, that point is considered a core point of the cluster. Points that fail to meet this criteria, but that are density reachable from a core point, are considered border points and are classified as part of the colony. Points that fail to meet either of these two criteria are labelled as noise and not part of any colony.

While there are implementations of DBSCAN that automate parameter optimization, these increase time complexity [35,36]. As an alternative to automatic parameter estimation, CE allows for the Eps and MinPts parameters to be adjusted via the graphical user interface while viewing the resulting colony identification accuracy. The immediate visual feedback enables intuitive and accurate colony classification and decreases the time it takes to optimize Eps and MinPts. DBSCAN clustering is deterministic for the core cells of each cluster and only border points which are density reachable from more than one cluster core can be assigned to different clusters between runs. Colonies in ECM patterned wells rarely grow close enough for border cells to be density reachable from more than one colony, so for this application cells are routinely clustered deterministically. To further increase colony identification accuracy, CE includes filters for colony size, density and roundness, which refine the colony identification procedure and are particularly useful to deal with imaging artefacts and overgrown colonies. These filters are controlled via sliders in the GUI and the resulting colony identification is immediately visualized for the selected well.

Each cluster of points returned by DBSCAN corresponds to cells growing together in a colony. To define colony attributes, CE utilizes the geometric analyses package Shapely [37]. Generally, the polygonal boundary area of a colony can be defined as either the convex or concave hull of its cells. CE uses the convex hull algorithm as it is less expensive to compute than the concave hull and performs well with commonly used micropatterned spot shapes. By finding the colony bounding area, additional geometric attributes such as colony diameter, circumference, area and cell density can be calculated for each colony. Additionally, each cell can be assigned Cartesian coordinates relative the colony’s centroid or the closest edge of the colony’s boundary area. These relative cell coordinates can be used to group cells at similar positions from multiple colonies into concentric bins. The process for grouping cells is initiated by deriving an aggregated value of all the cells in a colony that are within the same location bin. These colony values are then aggregated for multiple colonies and the error estimation reported describes the variation between colonies rather than between cells within one colony. The visualizations built into CE further facilitates the analyses of spatial trends within colonies. Cells can also be grouped according to a hexagonal grid, which aggregates cells from all colonies in the same bin within the grid.

## Results

### Colony classification

To demonstrate the colony classification process, we patterned mouse PSCs on circular ECM spots 500 μm in diameter in a 96-well plate using an in-house HTP UV-lithography method [21]. To enable extraction of cellular coordinates within the well, cell nuclei were labelled with DAPI and analysed by a primary image analysis program, such as CellProfiler. After imaging and extraction of single cell features, the resulting CSV-file was processed by CE to classify cells into colonies. Cells robustly adhered to the patterned regions, and were grown for 48 h in pluripotent conditions (LIF and Serum, see Supplementary Methods for detailed culture conditions). These micropatterned, well-separated clusters of cells were easily identified by CE as separate colonies. However, cells occasionally bridge adjacent confluent colonies, effectively merging two or more colonies together. Such colonies would still be classified as valid clusters by DBSCAN, since all the cells are density reachable from each other. Another important caveat is that the imaging hardware may not allow the entire well to be captured, resulting in partial images of many of the colonies. Including either of these merged or partial colonies in the downstream analyses could confound the interpretation of the underlying biology.

**Fig 2:**
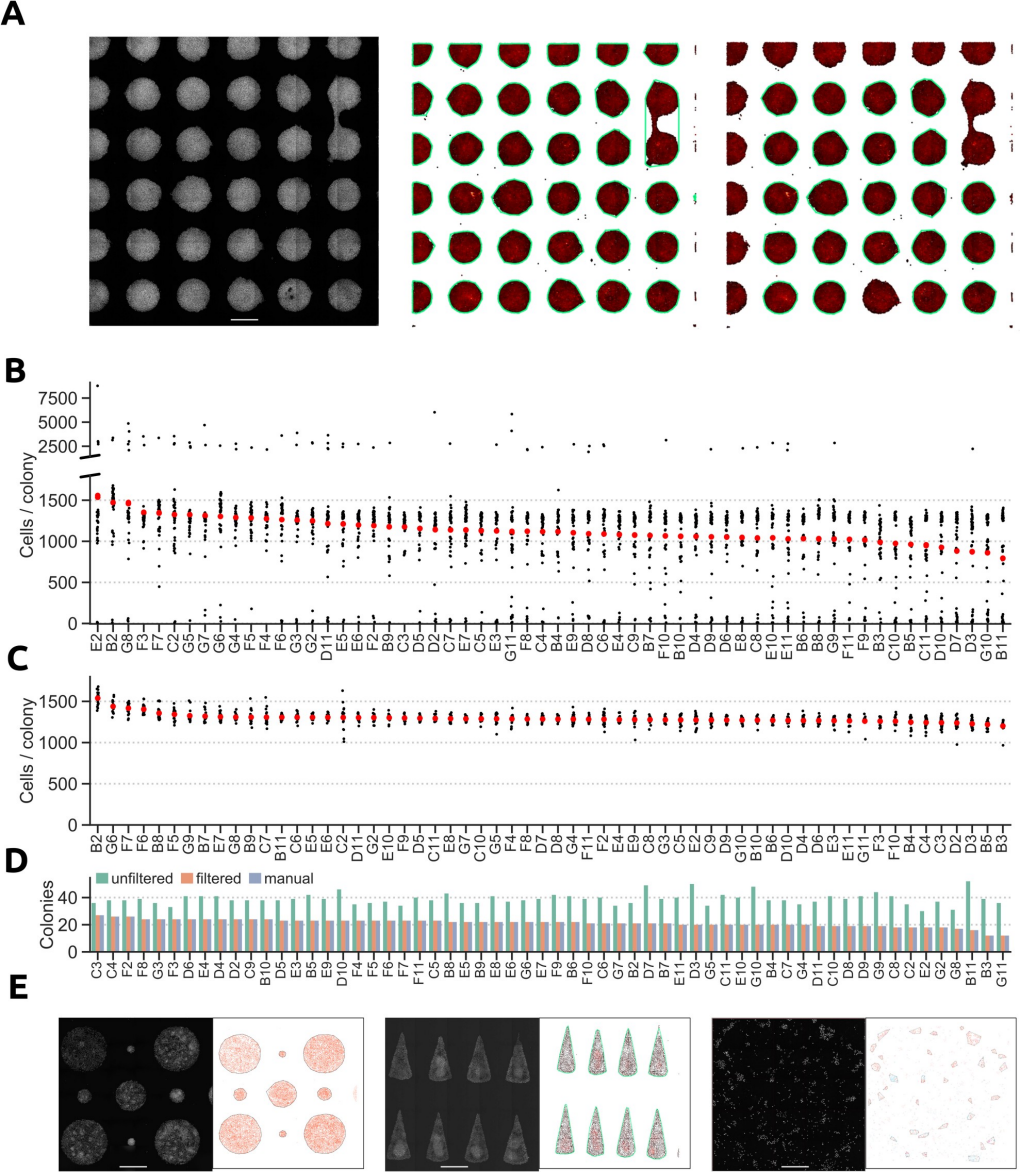
Classification of cells into colonies. **A)** mPSCs restricted to grow on micropatterned ECM spots (*left*) can be classified into colonies via the DBSCAN algorithm (*middle*). Merged and partial colonies can be excluded by applying filters to the DBSCAN clustering results (*right*). Each data point represents a cell and colonies are encircled with green lines. Scale bar = 500 μm. **B)** Number of cells per colony in wells from a 96-well plate after running DBSCAN. **C)** Number of cells per colony in wells after excluding merged and partial colonies from the DBSCAN results. **D**) Number of colonies per well after running DBSCAN with and without filters, and from a visual inspection of the images. **E**) Colony identification for colonies of different sizes in the same well (*left*), colonies of non-circular geometries (*middle*), and colonies in wells without pre-patterned ECM spots (*right*). Scale bar = 500 μm.

When limited to only the default DBSCAN parameters MinPts and Eps, partial and merged colonies are difficult to discriminate from colonies of desired shape and size. We found that an efficient way to eliminate these undesired colonies, was to apply a set of filtering criteria to the colonies detected by DBSCAN. Filtering on colony roundness and size were the most effective criteria to exclude merged and partial colonies from the DBSCAN output (**Fig 2A**). The effect of applying size and roundness filters was striking when comparing the number of cells per colony before and after filtering. Prior to filtering, several clusters of colony sizes were detected, including bigger merged colonies and smaller partial colonies cut off by the imaging limitations (**Fig 2B**). These colonies were omitted from the final analyses as they would skew the calculations of both the mean number of cells per colony and spatial trends within the colonies. After excluding colonies of undesirable size and shape, we observed only one cluster of colony sizes and the mean number of cells per colonies was notably consistent between wells, indicating reproducible patterning of cells (**Fig 2C**). The result of the filtering in CE was almost identical to manually identifying merged and partial colonies (406 of 412 colonies were correctly classified, **S1A Fig and S1B Fig**).

Another useful metric for assessing patterning fidelity is the number of colonies per well, which is also computed by CE. Comparing the number of filtered and unfiltered colonies identifies wells containing many fused and partial colonies that were detected by DBSCAN, but then excluded by the filters. The filtered count was nearly identical to the counts obtained from visually inspecting the images from each well (**Fig 2D**), and were computed in a fraction of the time of manual counting. CE can also accurately identify clusters of cells growing in micro-patterned ECM spots of different sizes within the same well, cells patterned in non-circular colony shapes, and colonies in unpatterned wells (**Fig 2E**). The flexibility of the parameter and filter adjustments makes it possible to identify colonies within unpatterned wells with high accuracy (the mean difference from a manual colony count was 2.6% (sd 1.3%) for ten wells with around 100 colonies in each, **S1C Fig and S1C and S1D Fig**). These results demonstrate the capacity for CE to semi-automatically identify colonies of a wide array of geometries both in patterned and unpatterned wells with an accuracy similar to that of visual image inspection.

### Investigating the behavior of hPSCs in micropatterned colonies

**Fig 3:**
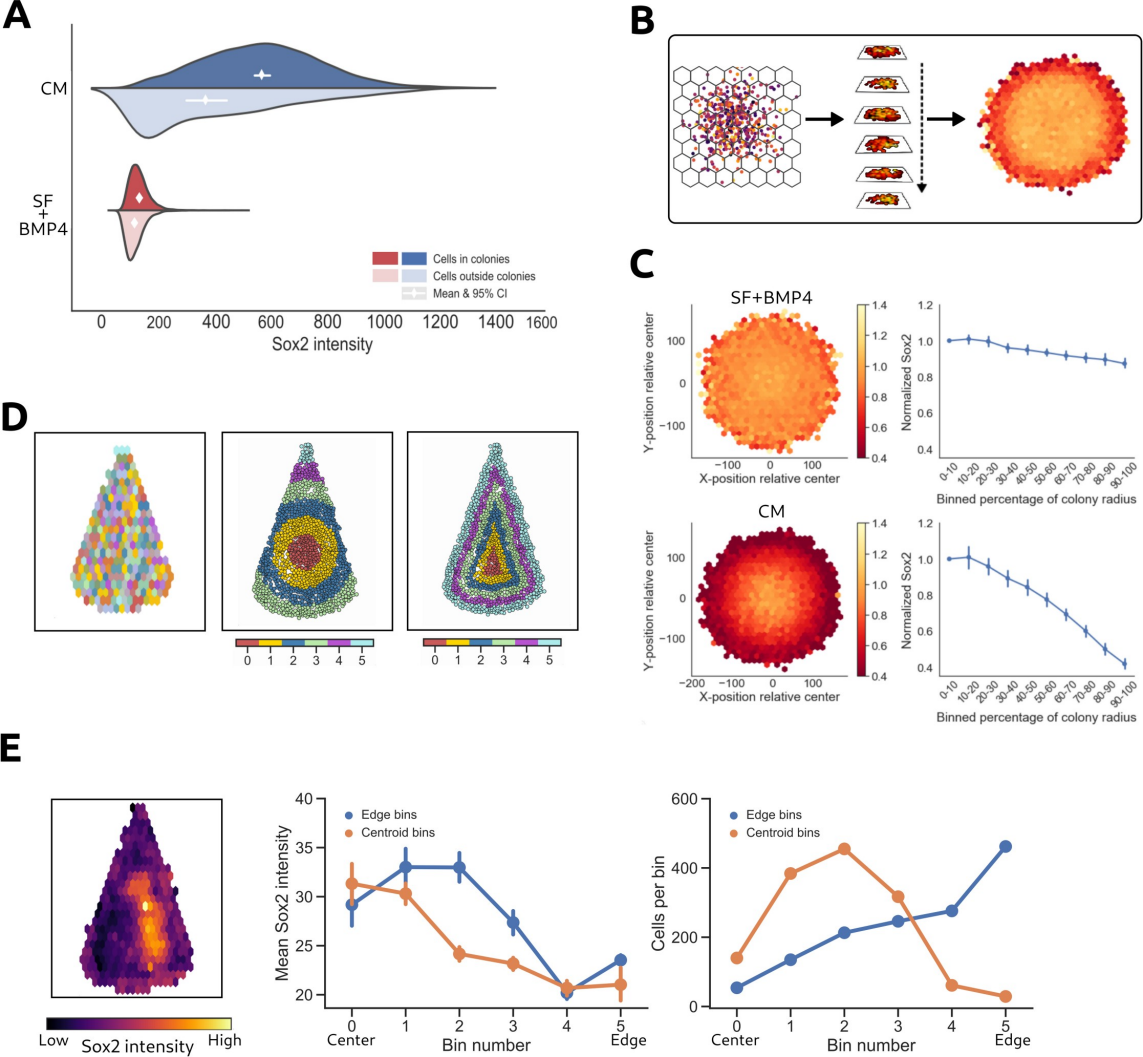
Quantification of radial expression trends. **A)** Comparison of SOX2 expression between cells growing inside and outside of colonies in microcontact-printed wells. **B)** Cells from multiple colonies at similar locations within their respective colony are aggregated together in bins according to a hexagonal grid system. The heatmap is colored by the desired measure of variation or central tendency. **C)** Multiple colonies of hPSCs are aggregated to reveal general tendencies in the spatial protein expression pattern of SOX2 (*left*). Each bin shows the mean expression of cells from multiple colonies. Trends are visualized as line plots, where joint data points represent the mean intensity of cells at each distance bin throughout the colony (*right*). Error bars represent 95% confidence intervals between cells from the same location in different colonies. **D)** Hexagonally binned cells in triangular hPSC colonies (*left*). Cells grouped in concentric bins according to their distance from the colony centroid (*middle*) or to the closest colony edge (*right*). The color is used for illustration purposes to distinguish bins from each other. **E)** Visualization of the Sox2 expression levels in the hexagonal bins (left) and the concentric bins (middle). The number of cells per bin for the different concentric binning strategies (right).

To apply CE to hPSCs analysis, we first patterned hPSCs in 200 μm diameter colonies in 96-well plates using microcontact-printing. While most cells adhere to the ECM spots in the patterned plates, there is also limited non-specific cell adhesion in-between patterned ECM spots. Compared to UV-lithography, microcontact printing has a higher proportion of cells growing in tiny colonies and as single cells outside patterned ECM spots, which makes this technology suitable for comparing the behavior of cells outside and inside micropatterned colonies. To test whether cells that adhere non-specifically display differences in protein expression compared to cells within colonies, we assessed cellular response 42 h after treatment with serum free medium containing BMP4 (SF+BMP4, induces trophectoderm and primitive endoderm [38,39]), or MEF conditioned medium (CM, maintains pluripotency [40]). Expression of the pluripotency-associated transcription factors SOX2 and OCT4 was analyzed to quantify cellular differences. As expected, in SF+BMP4 medium, pluripotency signals were repressed and no difference was observed in SOX2 and OCT4 expression between cells inside and outside colonies (**Fig 3A** & **S1E Fig**). In contrast, CM induces the expression of SOX2 and OCT4 in cells within colonies, while cells outside colonies express the marker to a lesser extent (**Figs 3A** & **S1E**). This is visible both as a change of shapes and a shift in means of the protein expression distributions. These differences suggest that cells inside and outside colonies do not respond similarly to added factors in the medium, further highlighting the importance of controlling for microenvironmental parameters such as population context when assessing cellular responses to experimental conditions.

### Analysis of spatial trends in protein expression within hPSC colonies

In addition to facilitating inter-colony analyses, CE allows for investigation of intra-colony variation in protein expression. Radial gradients of protein expression have previously been reported in non-patterned and patterned colonies of hPSCs [2,6,21,41]. Analysing these trends through visual inspection and manual data analyses is feasible in a low throughput platform, but becomes error-prone and time-consuming in HTP systems with hundreds or thousands of colonies. To visualize spatially biased protein expression, CE can automatically aggregate colonies within replicate wells and display a heatmap of spatial protein expression variation within these colonies (**Fig 3B**). This analysis can be applied to colonies of various sizes and shapes.

When investigating colonies of hPSCs grown in either CM or SF+BMP4, we observed distinct radial gradients of SOX2 and OCT4 expression between the two conditions (**Figs 3C** & **S1F**). To distinguish differences attributed to local spatial factors from those attributed to exogenous factors, all intensities were normalized relative to the expression at the centroid of the colony. In hPSCs grown in CM, SOX2-expression decreased in a linear fashion towards the edge of the colony, with cells at the colony border only displaying half the fluorescence intensity of cells near the colony centroid. Meanwhile, cells grown in SF+BMP4, exhibited low SOX2 expression throughout the entire colony, which can be attributed to BMP4 inducing differentiation and overriding any local pluripotency supporting signals. OCT4 expression followed similar patterns in both CM and SF+BMP4.

Quantitative evaluation of protein expression levels relative to the location of a cell within a colony was performed by aggregating cells in radial bins according to their distance from the colony centroid rather than their relative xy-coordinates. The mean or median expression values could then be compared between ring-shaped bins across multiple colonies. This analysis technique highlights the spatial trends of SOX2-expression for hPSCs grown in CM (**Fig 3C**), where expression of SOX2 was highest in cells at the center of the colony and linearly decreased toward the colony edge. Statistical significance at p = 0.01 can be roughly inferred from non-overlapping pairs of 95% confidence intervals [42]. However, it should be noted that statistical significance is easily achieved with sample sizes this large, even at small effect sizes [43], so it is important to assess the magnitude of the differences. In hPSCs cultured in SF+BMP4, a weak radial gradient of SOX2 expression emerged exhibiting no more than a 10% difference in expression level between cells at the centroid and the edge of the colony. In contrast, hPSCs cultured in CM exhibit more than double the level of SOX2 expression at the center of the colony compared to the edge.

For cells grown on ECM patterns of non-circular geometries, we evaluated how grouping cells into bins based either on the distance from the colony border or the colony centroid affected our interpretation of spatial trends in protein expression. To illustrate the different biological interpretations that could arise from these two metrics, we investigated the SOX2 expression of hPSC colonies grown on triangular ECM patterns in SF+BMP4.

In addition to aggregating cells in hexagonal bins as previously described, colonies were segmented into concentric annular or colony-shaped (in this case triangular) bins (**Fig 3D**). The annular and triangular segmentations were created based on the distance from the colony centroid or the closest colony border, respectively. When the intensity was visualized based on the hexagonal binning strategy a clear spatial bias in SOX2 expression was reveled (**Fig 3E**). To further quantify this organized expression, the average expression levels of cells grouped according to the annular and triangular bins was compared. Importantly, the choice of binning metric could influence the interpretation of the resulting protein expression trends. In this example, SOX2 expression decreased more rapidly as a function of the distance from the colony centroid compared to from the colony edge (**Fig 3E**). Depending on the binning strategy, cells were grouped to different bins and the number of cells in each bins differed greatly (**Fig 3E**).

### Conclusions

There is overwhelming evidence that increased control and monitoring of population context parameters is needed to improve assay reproducibility and to understand heterogeneous responses between cells in the same population. However, addressing this challenge has proven difficult in the broader biomedical community. To augment the power of HTP analysis of population context parameters in the cellular microenvironment, we previously developed cell patterning techniques to control population context parameters, and here we demonstrate a software tool for improved monitoring of microenvironmental variables and interrogation of community driven cell fate acquisitions in HTP assays. In this study, CE was utilized to explore and quantify radial spatial trends in SOX2 and OCT4 expression within micropatterned hPSC colonies of various shapes and sizes. We observed that the protein expression levels vary as a function of cells’ location within a colony, further highlighting the importance of understanding variation in population context dependent factors.

## Availability and future directions

CE is compatible with existing HTP imaging software and standard fluorescent microscopy based assays. By developing a GUI-driven workflow and releasing it under an open source license, we provide a solution to facilitate colony-level analysis for a wide scientific community. Members of our group regularly use CE for colony level analyses as evidenced in previously published and ongoing studies [19–21,44]. To further broaden the utility and applications of the software, there are built-in visualizations to assist with fluorescent intensity thresholding and pattern fidelity assessment, and there are interface components for assigning wells to treatment groups. To lower the threshold for wide adoption, CE is distributed as a Python package through the conda and pip package managers, and runs under Linux, OS X and Windows. The setup process does not require any use of the command line and can be done entirely from the Anaconda Navigator GUI. The source code is distributed under the open source BSD 3-clause license, which enables incorporation of its features into existing image analysis pipelines. Source code and installation instructions are available online at *https*:*//gitlab*.*com/stemcellbioengineering/context-explorer*, and the documentation can be found at *https*:*//contextexplorer*.*readthedocs*.*io*.

## Supporting information

S1 Text Supplementary methods. (DOCX)

S1 Fig Quantification of colony count precision and OCT4 expression data. **A)** 412 colonies from ten wells plotted according to their roundness and the number of cells in each colony. Misclassified colonies are highlighted with differently shaped and colored scatter markers. **B)** Quantification of the number of correctly and incorrectly classified colonies in panel A. **C)** CE colony identification in an unpatterned well with clustering parameters optimized for small colonies (left), large colonies (middle) or a mix of small and medium sized colonies (right). **D)** Well-wise comparison of the number of colonies identified by manual count or automatically by CE. The mean difference in the count was 2.6% (sd 1.3%). **E)** Differences in OCT4 expression level among cells inside or outside colonies. **F)** Hexbin and line plot averages for OCT4 expression levels in SF+BMP4 and CM.
(TIF)
