# Supplementary material for: Context-explorer: Analysis of spatially organized protein expression in high-throughput screens
Source: PLoS Comput Biol. 2019 Jan 2;15(1):e1006384. doi: 10.1371/journal.pcbi.1006384 (PMC6331134; doi:10.1371/journal.pcbi.1006384)
Supplement: S1 Text — (DOCX) [file pcbi.1006384.s001.docx]

# Supplementary Methods

## Microcontact printing

We previously developed a method for patterning proteins in standard 96-well plates using microcontact printing [1]. The PDMS stamps were fabricated using standard soft lithography techniques, with the exception that liquid PDMS was cast into a Teflon mould before curing, allowing control of the shape of the PDMS stamp. Microcontact printing was carried out according to our previously published protocol [[2](#ref-peerani_patterning_2009)]. Briefly, the ECM solution (Matrigel diluted 1:30 in phosphate buffered saline) was deposited onto the patterned surface of ethanol sterilized PDMS stamps for 4 h at room temperature. Stamps were rinsed with ddH2O, dried gently with N2 gas, placed into tissue-culture treated 96-well plates, and incubated in the 96-well plates for 7-10 min in a humidity chamber (Relative humidity 55-70%). The stamps were then removed and substrates were passivated with 5% weight Pluronic F-127 (Sigma-Aldrich) in ddH20 for 1 h.

## UV lithography

We recently developed an alternative method to microcontact printing using UV-lithography to pattern proteins in standard 96-well [3]. Briefly, glass cover-slips were activated in a plasma cleaner and rinsed in ddH2O. Patterns of predefined size and shape were created on the cover-slip by photo-oxidizing select regions of the substrate using Deep UV exposure. The patterned slides were assembled to bottomless 96-well plates to produce plates with patterned cell culture surfaces. Prior to seeding cells onto the plates, the wells were activated with N-(3-Dimethylaminopropyl)-N-ethylcarbodiimide hydrochloride (Sigma #03450), and N-Hydroxysuccinimide (Sigma #130672) for 20 minutes. Before seeding cells, the plates were incubated with Geltrex (for hPSC) or 12.5 µg / ml fibronectin in gelatin (for mPSCs) and washed with ddH2O.

## hPSCs culture and seeding onto patterned substrates

We obtained the H9 hESC line (WA09) from the WiCell Research Institute. H9 cells were routinely cultured on feeder layers of irradiated murine embryonic fibroblast (MEF) feeders in knockout (KO)-Dulbecco’s modified Eagle’s medium (DMEM) (Invitrogen) with 20% KO-serum replacement (Invitrogen) (KO-DMEM) supplemented with 4 ng mL–1 FGF-2 (PeproTech). Cells were passaged at 1:4 to 1:6 split ratios every 4-5 days by dissociating colonies with 0.1% collagenase IV (Invitrogen) into small clumps. All cell line stocks were confirmed negative for mycoplasma contamination.

The hPSCs were dissociated using TrypLE for three min. TrypLE was inactivated by adding medium containing 20% KO-SR. Cells were centrifuged and resuspended in Nutristem hESC XF (Biological Industries #05-100-1A) and 10 µM ROCK inhibitor Y-27632 (Tocris). The SF medium contains DMEM/F12, 1x Nonessential amino acids, 50 U/mL Penicillin, 50 µg/mL Streptomycin, 10 µg/mL bovine Transferrin, 0.1 mM ß-Mercaptoethanol (all Invitrogen), 2% fatty acid-free Cohn’s fraction V BSA (Serologicals), 1x Trace Elements A, B & C (Mediatech), 50 µg/mL Ascorbic Acid (Sigma) and 7 µg/mL recombinant human insulin. Cells were seeded at 10^5^ cells per well (or as described in text) and incubated. After 6 h, cells were washed twice with PBS and incubated for another 42 h in fresh medium (either SF supplemented with factors or CM).

## mPSCs culture and seeding onto patterned surfaces

For culture and experiments, R1 mPSCs were kept in media containing 15% heat-inactivated FBS (900-108, Gemini), 500 pM LIF (R&D systems #8878-LF-025), 100 AM 2-mercaptoethanol (Gibco #21985023), 2 mM L-glutamine (Gibco #21051024), 0.1 mM nonessential amino acids (Gibco #11140050), 1 mM sodium pyruvate (Gibco #11360070), 100 U/mL penicillin (Gibco #15140122), and 100 Ag/mL streptomycin (Gibco #15140022) in high-glucose DMEM (Gibco #11965092). In culture, cells were grown on gelatin-coated (0.2%) tissue-culture flasks and for experiments cells were grown on micropatterned 96-well plates coated with 12.5 µg / ml fibronectin in 0.2% gelatin. 50 * 10^4^ cells were seeded per well.

## Immunocytochemistry

Plates were fixed for 30 min in 3.7% formaldehyde and permeabilized for 3 min in 100% methanol. Next the plates were incubated overnight at 4°C with primary antibodies (SOX2 (1:400, R&D Systems #MAB2018) and OCT4 (1:400, BD Biosciences #611203)) in 10% FBS in PBS. Finally, the plates were incubated with AlexaFluor secondary antibodies (1:200; Molecular Probes) and Hoechst 33342 (Sigma #861405) or DAPI (Sigma #D9542) for 1 h 20 min at room temperature in 10% FBS in PBS.

## High-content image analysis

Plates were imaged and analyzed using the Cellomics Arrayscan VTI platform and Target Activation protocol (Thermo Scientific). This protocol generates nuclear masks, provides single cell average nuclear intensity values for protein expression and DNA content, as well as spatial xy-coordinates of the nuclei centroids. Single cell xy-coordinates and fluorescent intensity data were exported as CSV-files and imported into CE for exploration of colony level details.

## Statistical analysis

Error bars on plots represent 95% confidence intervals (CI) of replicates except where indicated differently. CIs are calculated by resampling the original distribution 1000 times as implemented in Seaborn.

## References

1. Nazareth EJP, Ostblom JEE, Lücker PB, Shukla S, Alvarez MM, Oh SKW, et al. High-throughput fingerprinting of human pluripotent stem cell fate responses and lineage bias. Nature Methods. 2013;10: 1225–1231. doi:[10.1038/nmeth.2684](https://doi.org/10.1038/nmeth.2684)

2. Peerani R, Bauwens C, Kumacheva E, Zandstra PW. Patterning mouse and human embryonic stem cells using micro-contact printing. Methods in Molecular Biology (Clifton, NJ). 2009;482: 21–33. doi:[10.1007/978-1-59745-060-7_2](https://doi.org/10.1007/978-1-59745-060-7_2)

3. Tewary M, Ostblom J, Prochazka L, Zulueta-Coarasa T, Shakiba N, Fernandez-Gonzalez R, et al. A stepwise model of Reaction-Diffusion and Positional-Information governs self-organized human peri-gastrulation-like patterning. Development. 2017; dev.149658. doi:[10.1242/dev.149658](https://doi.org/10.1242/dev.149658)
