# Supplementary figures and images for: Context-explorer: Analysis of spatially organized protein expression in high-throughput screens
Source: PLoS Comput Biol. 2019 Jan 2;15(1):e1006384. doi: 10.1371/journal.pcbi.1006384 (PMC6331134; doi:10.1371/journal.pcbi.1006384)

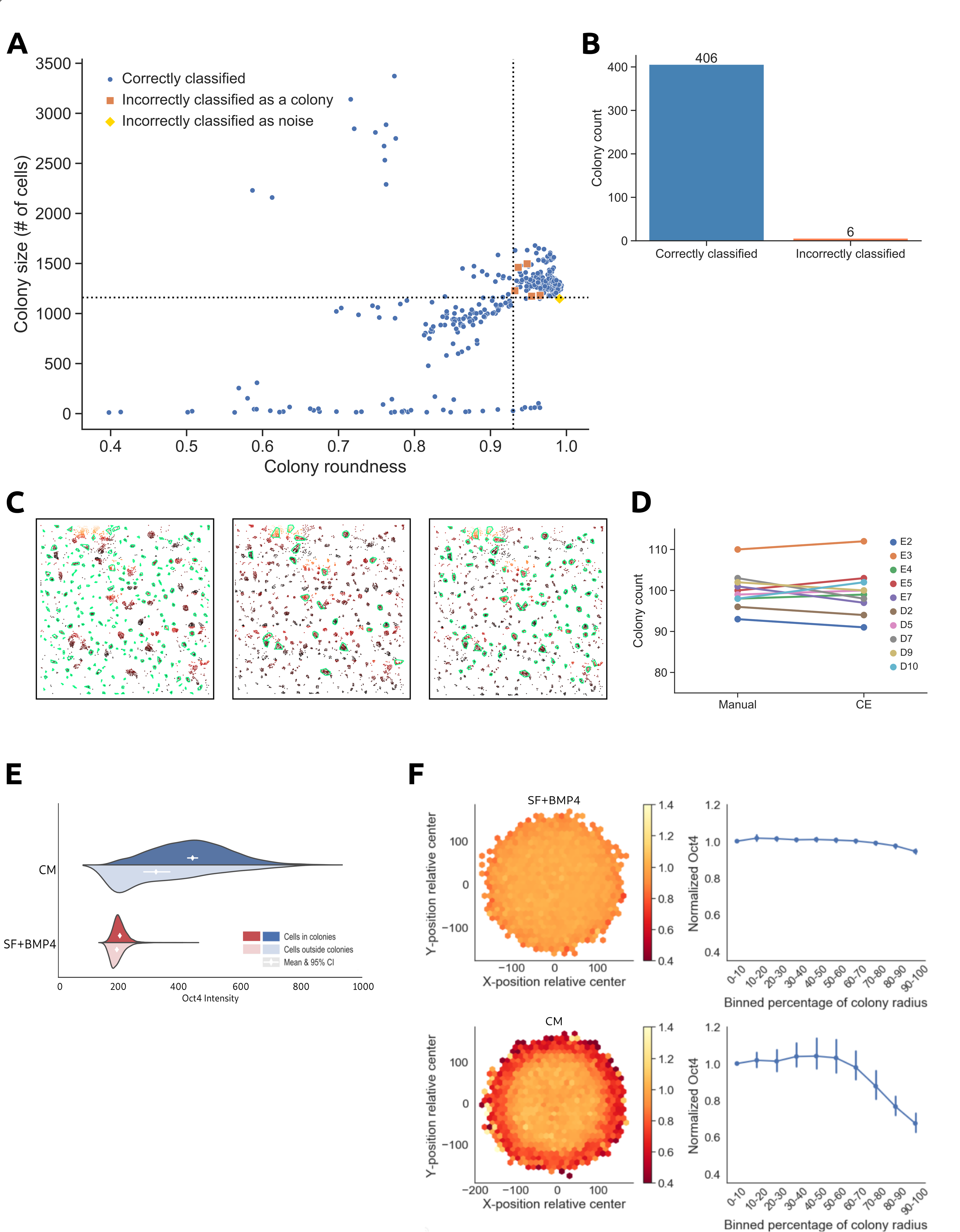

Supplement: S1 Fig — A) 412 colonies from ten wells plotted according to their roundness and the number of cells in each colony. Misclassified colonies are highlighted with differently shaped and colored scatter markers. B) Quantification of the number of correctly and incorrectly classified colonies in panel A. C) CE colony identification in an unpatterned well with clustering parameters optimized for small colonies (left), large colonies (middle) or a mix of small and medium sized colonies (right). D) Well-wise comparison of the number of colonies identified by manual count or automatically by CE. The mean difference in the count was 2.6% (sd 1.3%). E) Differences in OCT4 expression level among cells inside or outside colonies. F) Hexbin and line plot averages for OCT4 expression levels in SF+BMP4 and CM. (TIF) [file pcbi.1006384.s002.tif]
